# Organisational and social work-environment experiences after care manager implementation: a repeated cross-sectional study in Swedish primary care

**DOI:** 10.1080/02813432.2025.2538486

**Published:** 2025-07-28

**Authors:** Pia Augustsson, Eva-Lisa Petersson, Cecilia Björkelund, Sven Persson Kylén, Carl Wikberg

**Affiliations:** ^a^ General Practice/Family Medicine, School of Public Health and Community Medicine, Institute of Medicine, Sahlgrenska Academy, University of Gothenburg, Gothenburg, Sweden; ^b^ Region Västra Götaland, Research, Education, Development and Innovation (REDI), Primary Health Care, Gothenburg, Sweden

**Keywords:** Primary health care, care manager, personnel, organisational and social work environment, Collaborative care

## Abstract

**Introduction:**

Primary care centers (PCCs) are the foundation of healthcare, requiring a supportive work environment for quality care and personnel well-being. To address rising common mental disorders (depression, anxiety, stress-related disorders) care managers were introduced in Region Västra Götaland in 2015 and are now established in 175 PCCs, supported by clinical and economic benefits. This study explores changes in the organisational and social work environment experienced by PCC personnel five years post-implementation of care managers at two points: 2016/17 and 2021/22.

**Materials and methods:**

This repeated cross-sectional study was conducted as an open cohort at 36 strategically selected PCCs in 2016/17 and 2021/22. Although the personnel varied, consistent instruments were used. Descriptive statistics and cross-tabulations examined differences in the organizational and social work environment at the two periods.

**Trial registration:**

NCT02378272, 2015-02-02. Retrospectively registered.

**Results:**

Familiarity with the care manager function increased from 72% in 2016/17 to 79% in 2021/22. Motivation to collaborate increased by 80%, reflecting a 62% higher willingness compared to 2016/17. Personnel’s knowledge and motivation were significantly associated with perceived improvements in the work environment. These improvements were consistent across gender, age, PCC size, and geographic location. Personnel at smaller PCCs reporting slightly greater improvements, with some age-related variation.

**Conclusions:**

Having a care manager on site may have limited direct impact on individual work or perceptions of the work environment. However, increased knowledge of the function appears to strengthen collaboration collegial support within the PCC, indicating positive changes in the social and organisational work environment.

## Introduction

Primary care centers (PCCs) serve as the foundation of healthcare systems worldwide, providing accessible, first-line care for a wide range of health concerns [1,2]. As healthcare systems face increasing demands due to rising prevalence of common mental disorders (CMD) such as depression, anxiety and stress-related disorders along with growing multimorbidity and workforce shortages, a supportive work environment is essential for maintaining high-quality patient care and ensuring the well-being of healthcare professionals [3–5]. However, primary care settings are often considered high-risk environments for poor working conditions, leading to work-related stress and health issues among professionals [6–8].

Collaborative care interventions with care managers are organizational strategies designed to improve patient care through leadership and decision support and can be implemented in various healthcare settings, including PCCs [9,10]. The care manager puts collaborative care into practice by implementing integrated, team-based approaches that facilitate interprofessional communication and structured care planning for the patients [9,11]. The care manager’s function includes improving access to and continuity of care through direct patient contact [11], as well as promoting educational development within the PCC to improve communication and feedback both within the PCC team and with secondary care [8,12–14].

To address the rising prevalence of CMD, Region Västra Götaland introduced care managers in 2015 with the aim of improving care quality for individuals with CMD [15,16]. Today, 175 PCCs have trained one or more personnel members, e.g. nurses, district nurses, counselors, or psychologists, and implemented the function, an initiative supported by national guidelines that recommend continued expansion towards nationwide implementation [12].

Randomized controlled trials have demonstrated multiple benefits of care managers, including improved patient recovery [16], increased consistency in antidepressant medication use [12,17], and positive societal health economic outcomes [14]. Despite these benefits, integrating and sustaining the care manager function within PCC is a challenge and requires active engagement from the entire healthcare team. Changes in the work environment, such as introducing new professional functions, often bring challenges that can affect the work environment. These challenges may include increased workloads, shifts in the team, and the need for new collaborative structures [18–22].

Research has demonstrated a strong association between workplace quality and overall job satisfaction, even during periods of organisational change [5,23]. A supportive work environment not only promotes high-quality patient care but also reduces workplace stress and improves healthcare professionals’ well-being [4,24]. Furthermore, patient-centered and team-based approaches have been associated with higher job satisfaction and a more positive work climate with reduced workplace stress [25,26]. However, there is limited knowledge about how new functions, such as care managers, influence the work environment in PCCs over time. Organisational changes can introduce challenges, such as additional tasks, time pressures, and varying team perspectives, which may hinder collaboration and impact job satisfaction [27–29].

This repeated cross-sectional study addresses this knowledge gap by exploring the long-term changes in the work environment following the implementation of care managers in Swedish PCCs. By analyzing these changes, the study seeks to provide insights into how an implementation of organisational strategies can influence healthcare professionals and their working conditions, ultimately contributing to the development of sustainable and supportive workplace environments.

The aim of this study was to explore changes in the organisational and social work environment experienced by PCC personnel in a five-year period following the implementation of care managers.

## Materials and methods

### Study design and setting

A repeated Cross-Sectional study conducted as an open cohort at PCCs in the Region Västra Götaland in 2016/17 and again in 2021/22 [30]. This approach allows us to observe change in the work environment at PCCs following the implementation of care managers. The open cohort design implies that, while the same PCCs participated, the personnel involved may have varied over time. It should be noted that the second part of the data collection occurred toward the end of the COVID-19 pandemic. While the pandemic is not the focus of this study, it may have influenced participants’ responses regarding their work environment.

The study was approved by the regional ethical review board in Gothenburg, Sweden, and the Swedish Ethical Review Authority. The study was conducted according to the 1964 Declaration of Helsinki.

### Participants

In the initial phase of this repeated cross-sectional study conducted in 2016/17, 83 PCCs in Region Västra Götaland had an established care manager. From these, 39 centers were strategically selected to ensure a manageable, yet representative sample based on geographical and organisational differences. The strategic sampling process involved three steps: (1) identifying geographical locations, (2) categorizing areas into city, urban, or rural settings and distinguishing between small and large PCCs, and (3) further dividing them by private or public organisation within each area [30]. The participating PCCs are considered representative of the Västra Götaland region, suggesting that the results may be generalizable to other parts of Sweden or to countries with similar healthcare systems.

### Data-collection

The data was collected at two time points: from November 2016 through April 2017 [30], and from September 2021 until July 2022. In 2016, a digital survey (PCMQ I) was conducted, and email addresses were obtained from PCC directors collected from the Primary Healthcare Head Office, PCCs’ websites, and the primary care’s research and development unit (FoUUi).

As preparation for the follow-up survey in 2021/22, the email addresses of the 39 PCCs that were included in the study 2016 [30] were collected from the PCCs’ websites in September 2021. Directors were contacted by email, and sixteen directors provided email lists or permitted collection from personnel registers. The survey was administered from September 2021 to June 2022.

Both studies were administered by esMaker (https://entergate.se/products/esmaker/). A personal email was distributed to the personnel, with a link to the questionnaire and a cover letter with information about the studies purpose and terms of participation, according to the Declaration of Helsinki. During the data collection periods six reminders were sent by email at intervals. PCC directors in both surveys were asked to remind personnel at PCCs during the later part of the data collection periods.

### Questionary

The questionnaires were administered in 2016/17 and 2021/22. The first section of the questionnaires captured background characteristics, including gender, age, profession, and PCC name and funding (private or public).

The second section focused on the organisational and social work environment, addressing guidelines, collaboration motivation, changes in daily patient work, directors’ support, and clarity of responsibilities and priorities, in the context of the care manager function. Responses were measured using a five-point Likert scale (1 = "totally disagree, to " 5 = "totally agree"). In the 2021/22 questionnaire, questions from the Consolidated Framework for Implementation Research (http://www.cfirguide.org) [31,32] were utilized, categorizing them into two domains: the organisational work environment (e.g. management and governance, communication, participation, distribution of tasks, requirements, resources, and responsibilities) and the social work environment (e.g. social interaction, cooperation, and support from both directors and colleagues) as defined by The Swedish Working Environment Agency’s regulation AFS 2015:4 [33].

In the 2021/22 questionnaire, a third section was added to assess how external factors, such as the Covid-19 pandemic, influenced PCC personnel’s collaboration with the care manager in relation to the organisational and social work environment. Two Likert scales were used with a five-point scale where 1  =  “Very little extent” and” 5 = “Very large extent” and 10 points scale where totally agree 1  =  “Totally an impairment” and” 5 = “Totally an improvement”. (See supplementary file 1 - Questionary).

### Statistical analysis

Data analysis was conducted by statistical software SPSS version 29.0. Descriptive statistics were used to present the demographic characteristics of the participants. Item statistics were compiled for the following groups:

1. The study as a whole.
2. Professionals and administrative personnel, respectively.
3. Small and large (<24 employees or ≥24 employees) PCC units.
4. City (>100 000 residents), urban (town with >200 residents), and rural (area with villages with <200 residents) PCC units.

In the analyses of the repeated cross-sectional data, the response scale ranging from 1 to 5 were categorized into a 3-point scale, i.e. 1-2, 3, 4-5, and those on a scale 1-4 were dichotomized as a 2-point scale, i.e. 1-2, 3-4.

Chi-square tests were used for comparing frequencies, with all tests conducted as two-sided. Data with ordinal scale was analysed by Kruskal-Wallis and Mann-Whitney tests, while data measured on a continuous scale were analysed by ANOVA and Independent Samples t-Test.

Crosstabulation was used to statistically ensure if there were any differences between the groups by using the χ^2^-test. In cases where there were fewer than five respondents in cells, the Fisher-Freeman-Halton Exact Test was used.

## Results

**Figure 1.**
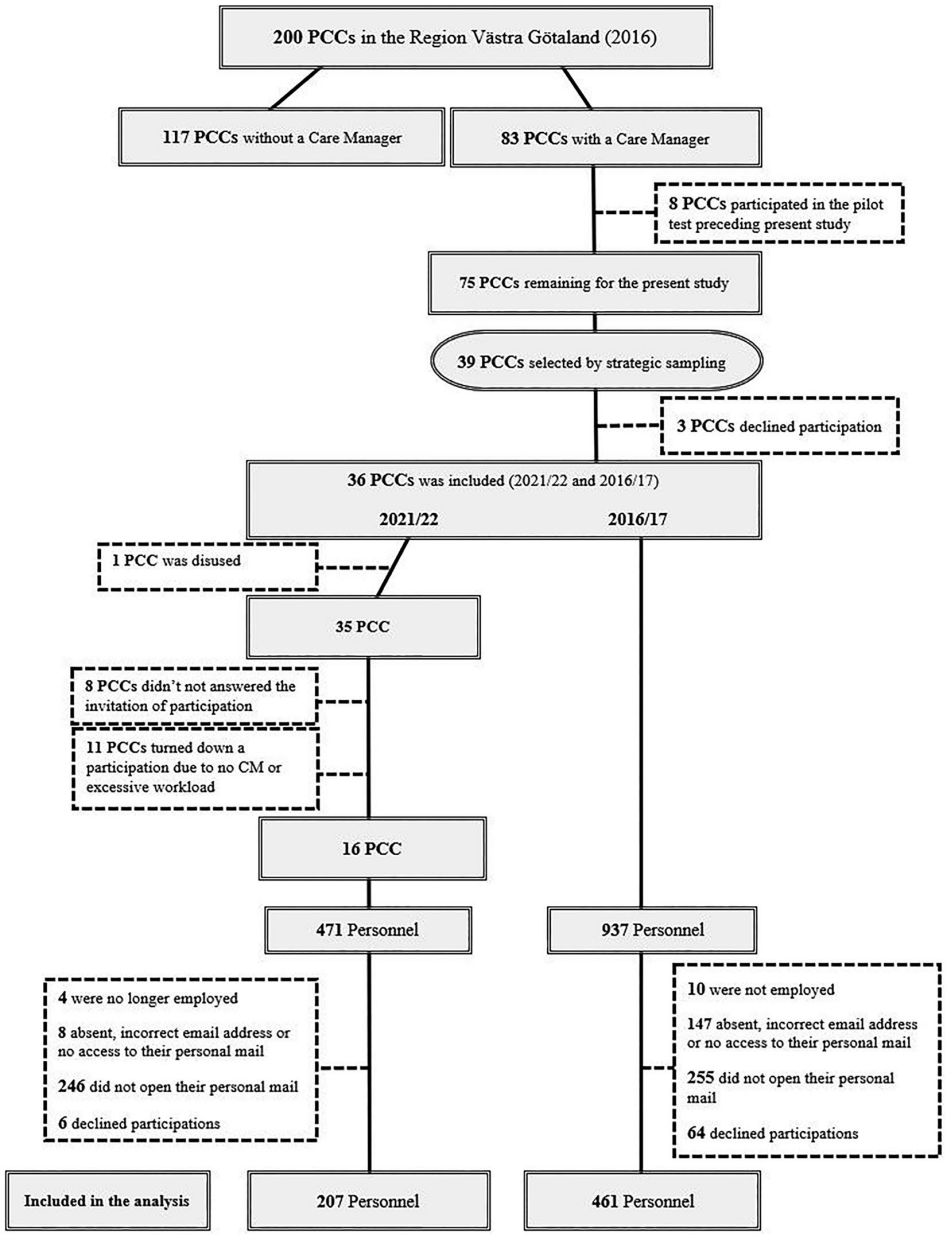
Flowchart of the inclusion procedure for the measurement points 2016/17 and 2021/22.

In this study, with two measure points 2016/17 and 2021/22, 39 PCCs were invited, Three PCCs declined participation, citing workload constraints. A total of 461 (56%) personnel from the selected centers participated in 2016/17. For the follow-up study in 2021/22, the same 36 PCC with personnel (clinicians and administrative personnel) as in 2016/17, were reinvited to conduct a follow-up questionnaire as part of this repeated cross-sectional study. One PCC was closed, 11 PCCs declined participation due to workload, and eight did not respond to the invitation. A total of 207 personnel participated in 2021/22 (See flowchart Figure 1).

### Participants’ characteristics

In both data collecting years (2016/17 and 2021/22), there were 86% females, 50% were in the age range of 31–50 year. When comparing the two data collection periods, supplementary subgroup analyses did not show systematic bias related to mix of profession, PCC size, or location. Overall, the background characteristics were proportionally similar despite the number of participants being halved, with differences of ≤10% with some exceptions. For instance, 15% more personnel from rural areas participated in 2021/22 compared to 7% in 2016/17. Most of the personnel, covering 74% in 2016/17 and 87% in 2021/22, were employed in larger PCCs with ≥24 employees, and the most common geographical location was urban.

Personnel in care-providing professions were the most represented, with 89% in 2016/17 and 80% in 2021/22, with nurses being more common, followed by physicians. For further presentation of the descriptive statistics (see Table 1).

**Table 1.** Background characteristics of the study populations in 2016/17 and 2021/22.

	2016/17	2021/22	
	Personnel, *n* = 461	Personnel, *n* = 207	*P* Value
Gender	*n* = 444	*n* = 207	
Female, n (%)	380 (85.6)	177 (85.5)	
Male, n (%)	64 (14.4)	30 (14.5)	n.s.
Age	*n* = 452	*n* = 207	
20–30, n (%)	32 (7.1)	15 (7.2)	
31–50, n (%)	228 (50.4)	103 (49.8)	
51-, n (%)	192 (42.5)	89 (43.0)	n.s.
Personnel	*n* = 453	*n* = 207	
Public, n (%)	322 (71.1)	158 (76.3)	
Private, n (%)	131 (28.5)	49 (23.7)	n.s.
Employment	*n* = 449	*n* = 187	
Care providing^1^, n (%)	398 (88.6)	163 (85.6)	
Administrative^2^, n (%)	51 (11.4)	24 (14.4)	n.s.
Profession	*n* = 449	*n* = 207	
Physician, n (%)	134 (29.1)	54 (26.1)	
Nurse, n (%)	153 (33.2)	55 (26.5)	
Assistant nurse, n (%)	38 (8.5)	17 (8.3)	
Psychosocial health^3^, n (%)	30 (6.7)	27 (13.2)	
Administrative^4^, n (%)	40 (8.9)	14 (6.9)	
Other^5^, n (%)	51 (11.4)	19 (9.4)	.001
PCC size	*n* = 406	*n* = 188	
<24 Personnel, n (%)	104 (22.6)	31 (15.0)	
≥24 Personnel, n (%)	302 (74.4)	157 (87.2)	.026
PCCs’ geographical locations^6^	*n* = 406	*n* = 188	
City, n (%)	127 (31.3)	80 (38.9)	
Urban, n (%)	195 (48)	80 (38.9)	
Rural, n (%)	84 (20.7)	28 (35.1)	.029

Note: Chi-square tests from multiple crosstabs. Degrees of freedom vary by comparison.

^1^ Physician, nurse, assistant nurse, psychologist, social worker, physiotherapist, rehabilitation coordinator, chiropodist, or occupational therapist.

^2^ Director, medical secretary, receptionist, biomedical analyst, economist, team leader or human resources.

^3^ Psychotherapist, social worker or psychologist.

^4^ Medical secretary or receptionist.

^5^ Physiotherapist, coordinator of rehabilitation, chiropodist, occupational therapist, biomedical analyst, economist, caretaker, or human resources.

^6^ City location: >100 000 residents, Urban location: Town with ≥200 residents, Rural location: Area with villages with <200 resident.

Table 2 presents the changes in how the personnel answered statements concerning the care manager function in 2016/17 and 2021/22. The results show that the personnel’s knowledge about the care manager function and motivation to work with the care manager together significantly affected their experience of the organisational and social work environment. However, there was an inconsistent picture of the changes in the personnel’s experiences, which were mixed positive and negative in both organisational and social work environment.

**Table 2.** Positive and negative changes in personnel responses concerning the care manager function at the measurement points 2016/17 and 2021/22.

	Completely/partly disagree		Neither agree nor disagree		Almost/completely agree			
		
	% (n)	% (n)	% (n)	% (n)	% (n)	% (n)		
CFIR-constructs	2016/17	2021/22	2016/17	2021/22	2016/17	2021/22	P value*	Changes
Organisational Work Environment								
Domain 2: Outer Setting								
*Patient needs and resources*								
Working with care manager has low priority	67.2 (197)	57.1 (101)	17.4 (51)	27.7 (49)	15.4 (45)	15.3 (27)	0.026	Positive
The care manager’s function has led to a noticeable change in my work with treating patients	32.4 (100)	33.0 (58)	26.5 (82)	30.1 (53)	41.1 (127)	36.9 (65)	NS	
*External policies and incentives*								
Working with care coordination has high priority	13.3 (39)	30.5 (54)	26.5 (78)	24.9 (44)	60.2 (177)	44.6 (79)	NS	Negative
Domain 3: Inner setting								
*Readiness for Implementation*								
At my PCC there are routines and/or guidelines for the care manager function	15.0 (67)	20.8 (37)	23.7 (10)	30.9 (55)	61.3 (274)	48.3 (86)	0.012	Negative
The PCC has a clearly formulated goal regarding the care manager function	27.4 (122)	20.9 (37)	47.5 (212)	31.1 (55)	25.1 (112)	48.0 (85)	<.001	Positive
Lack of clarity regarding what the care manager function entails	61.3 (214)	46.9 (83)	14.9 (52)	27.7 (49)	27.7 (83)	25.4 (45)	<.001	Negative
Lack of clarity regarding the distribution of responsibility between myself as a personal member and the care manager	63.3 (181)	53.7 (95)	17.5 (50)	22.6 (40)	19.2 (55)	23.7 (42)	NS	
Social Work Environment								
Domain 3: Inner setting								
*Networks and communications*								
I can collaborate with the care manager without encountering any problems	12.1 (37)	13.6 (24)	12.1 (37)	23.2 (41)	75.8 (232)	63.3 (112)	0.004	Negative
I have support from colleagues regarding my cooperation with the care manager	5.7 (27)	16.4 (29)	19.6 (51)	20.3 (36)	70 (182)	63.3 (112)	NS	
I have support from the PCC director regarding my cooperation with the care manager	5.7 (16)	18.1 (32)	15.9 (45)	20.3 (36)	78.4 (222)	61.6 (109)	<.001	Negative
Domain 4: Characteristics of Individuals								
*Knowledge and beliefs of the Intervention*								
I have low knowledge about the care manager function	32.4 (146)	41.2 (73)	19.8 (89)	25.4 (45)	47.8 (215)	33.3 (59)	0.005	Positive
I am motivated to collaborate with the care manager at my PCC	7.8 (35)	7.3 (13)	74.4 (334)	12.4 (22)	17.8 (80)	80.2 (142)	<.001	Positive

CFIR, Consolidated Framework for Implementation Research; NS, Non-significant.

* Significant difference p= <0.005.

### Organisational factors

In 2021/22, 79% of personnel had knowledge about the care manager function at their PCC, reflecting a 7% increase from 2016/17. Female personnel exhibited increased awareness from 70% to 79%. Among those with knowledge, 59% agreed that established routines and guidelines existed, whereas only 3% without knowledge believed in their existence, indicating a substantial decrease of 65% compared to 2016/17.

The analysis further revealed that among personnel aware of the care manager function, 53% were more unsure in 2021/22 about having sufficient knowledge, marking a 10% decrease from the previous period. There was no significant difference in personnel’s experience of engaging with care managers or in their patient work between 2016/17 and 2021/22.

The results also show differences among personnel in their experience of the balance between resources and demands at the PCC, particularly during the working day. Key findings are highlighted in supplementary files 2 and 3.

### Social factors

Regarding the factors in the social work environment the results show that personnel were 80% more motivated to work with the care manager in 2021/22, reflecting a 62% increase in willingness to collaborate compared to 2016/17. These positive changes remained consistent across various demographics, including gender, employment status, age, PCC size, geographic location, and public/private PCC. Among the professionals the physicians had the largest increase between the two measure points (2016/17 vs. 2021/22) in motivation (11% vs. 84%), close behind were the nurses (11% vs. 70%), the psychosocial health personnel (7% vs. 96%), and other personnel (39% vs. 88%) (see Table 2).

Observing in relation to collaboration with the care manager, 75% of personnel with knowledge about the care manager felt supported by their colleges in their collaboration with the care manager. At the same time, there was a 10% decrease in support from directors among personnel with knowledge of the care manager function. However, this decrease was higher among personnel without such knowledge, with a significant 30% decrease in support from directors.

### Influence of covid-19 on the collaboration with the care manager

Analysis of the data indicates significant age-related differences in the perceived difficulty of working with care managers during the COVID-19 pandemic in 2021/22. Additionally, personnel at small PCCs reported moderately higher levels of positive changes in the overall organisational work environment compared to those at large PCCs during the pandemic.

The results also revealed subtle differences in experiencing support during COVID-19, with professionals reporting slightly higher levels than administrative personnel, confirmed in separate analyses (see Table 3).

**Table 3.** COVID-19’s influence on the social and organisational work environment.

		Clinicians			Administrative personal			
		n	Mean	(SD)	n	Mean	(SD)	*P*
The social work environment during the COVID −19 pandemic	There was support for work situation from your director	139	3.97	1.161	20	3.15	.821	.004
	There was support for work situation from your colleges	139	4.03	1.191	20	3.16	.879	.003
		Small PHCC			Large PHCC			
		n	Mean	(SD)	n	Mean	(SD)	*P*
The organisational work environment during the COVID −19 pandemic	The pandemic has contributed to an improved social work environment for you	28	2.75	1.266	128	1.112	.445	.047
	The pandemic has contributed to an improved organisational work environment for you	28	2.82	1.09	128	2.31	.509	.014

Furthermore, professionals reported higher perceived support from colleagues during COVID-19, validated in smaller professional groups. Additionally, subtle yet significant distinctions between small and large PCCs were found in COVID-19’s impact on organisational and social work environments.

## Discussion

This study explored changes in the organisational and social work environment experienced by PCC personnel, comparing the measurement points 2016/17 and 2021/22, with a specific focus on the post-implementation and integration of the care manager function. Our findings suggest that increased knowledge about the care manager function was associated with greater motivation to collaborate with the function, and with more positive perceptions of work routines and collaborative care.

The care manager was introduced to strengthen care for patients with CMDs, but the implementation of such a function may also have influenced internal groups and perceived support within the PCCs. This has been confirmed in several studies examining the implementation and practical work of care managers, as well as how specific professional groups, such as physicians, have experienced collaboration with care managers from various perspectives [16,21,34,35].

Notably, we observed a decrease in perceived support from both colleagues and directors, particularly among those without knowledge of the care manager function. One possible explanation is that the introduction of care managers, as coordinators of mental health care, shifted certain responsibilities, such as patient follow-up or team collaboration, away from directors or other personnel. This reorganization may have changed perceptions of where support is created within the team [27,28].

It is also important to acknowledge that not all professionals at a PCC work directly with patients with CMD. The relevance and visibility of the care manager function may therefore vary depending on professional background, everyday clinical practice and individual differences in coping with changes. This may partly explain the variation in perceptions across personnel categories. Future research should explore how different functions relate to the care manager function and how this influences experiences of collaboration and support [15,26,36].

While the care manager function appeared to be more integrated in 2021/22 compared to 2016/17, personnel did not report major changes in their daily work due to the function. This could reflect that the function had become a more routine part of PCC. However, the increased knowledge and motivation to collaborate suggest that the care manager had gained acceptability within the PCC over time, possibly supported by the structured regional implementation efforts, such as mandatory training and financial inducements [15,16,20,37].

We acknowledge that the study period included the COVID-19 pandemic, which brought major challenges to primary care. Although pandemic-related effects were not a primary focus of this study, the timing likely influenced perceptions of the work environment. For example, we observed more nuanced patterns of perceived support during the pandemic, with physicians and nurses reporting higher support from colleagues and directors compared to administrative personnel. These patterns were particularly visible in smaller PCCs [38]. Such contextual factors and age-related differences in COVID-19 experiences highlight the importance of considering function specific and organisational differences when evaluating perceived workplace changes [38–40].

Finally, while this study does not provide direct evidence of changes in the overall work environment, such as workload intensity or clarity in the distribution of responsibilities, whether social or organisational, related to the care manager function, previous literature has shown that poorly implemented organisational changes can lead to perceptions of increased work overload or resistance [20,40,41]. Our results instead underline the importance of a clear understanding of the care manager function and shared engagement among directors and personnel actively involved in decision-making during implementation processes, in order to foster a supportive and collaborative work environment [18–24,42].

### Strengths and limitations

This study addresses a knowledge gap by being one of the first in Sweden to examine the long-term effects following the implementation of care managers. It explores whether personnel perceive any changes in the organisational and social work environment at PCCs, focusing on the periods 2016/17 and 2021/22 post-implementation.

Another strength is the use of identical questions at both time points, ensuring comparability of responses. These questions were refined through a pilot study conducted in 2016/17 before their final distribution. A further strength is the longitudinal approach, in the form of a repeated cross-sectional design, as the study revisited the same PCCs over time. The demographic distribution remained consistent across both measurement periods, enhancing the representativeness of the sample despite its relatively small size. However, it is important to acknowledge that the ability to infer causal relationships remains limited due to the observational design and potential external influences over time.

There are several limitations to consider. A major limitation is the low response rate and dropouts at the PCCs between 2016/17 and 2021/22, with the response rate halving between these periods (see Figure 1). This drop in participation may have affected representativeness and internal validity of the results. Rather than providing a definitive picture, the findings should be interpreted as indicative of changes. This raises concerns about reliability, particularly in relation to whether the dropout rate was too high to draw firm conclusions. Future studies should therefore aim for higher response rates to strengthen generalizability and ensure more reliable and robust conclusions.

It is important to note that the impact of COVID-19 cannot be overlooked, even though it was not the primary focus of this study, the dropouts of both PCCs and individual personnel can be attributed to the COVID-19 pandemic, which created a significant barrier to participation. Several PCC directors declined involvement due to personnel shortages and pandemic-induced workloads, and a lack of available care managers limited participation at some PCCs.

Non-response may have introduced a negative bias, as individuals with more negative attitudes or limited collaboration with care managers might have been less likely to participate in the follow-up. Those who found the study irrelevant or held unfavorable attitudes of the care manager function may have chosen not to respond. Another explanation could be the persistently high workload in primary care, where responding to questionnaires is often deprioritized. Additionally, collaboration with care managers, whether primarily shaped by individual engagement or governed by organisational structures. may have influenced both participation and attitudes toward the function. These factors, along with personnel turnover (especially among physicians and nurses) and an overload of research questionnaires following the lifting of COVID-19 restrictions, may have further reduced the response rate.

It is important to consider potential biases introduced by factors such as newly trained or recently hired personnel, who may have different perspectives due to limited experience with the care manager. Similarly, PCCs that have not fully integrated the care manager function may show different team interactions, which could have influenced responses and perceptions of the work environment.

A methodological limitation of this study is the use of an unvalidated questionnaire. Although the questionnaire was developed based on previous research and reviewed by experts in primary care and work environment to ensure its contextual relevance to Swedish primary care, its psychometric properties remain uncertain despite pilot testing and modifications made in 2016/17. This limitation restricts the generalizability and strength of the conclusions, and therefore, the findings should be interpreted with caution. Nonetheless, the results suggest that care managers may have a positive impact on both the organisational and social work environment.

Future research should prioritize the development and use of validated, standardized instruments to improve measurement accuracy and comparability across studies and over time.

### Implication

In summary, our study provides valuable insights into how the implementation of care managers may influence the evolving work environments of PCCs. The increased changes in motivation and knowledge about the function suggest that a care manager has the potential to positively impact both the organisational and social work environment. This should be considered when discussing the benefits that care managers can bring to PCCs. However, it is also crucial to emphasize the importance of adhering to regulations, developing targeted strategies, and continuously improving the work environment to address the challenges in primary care [29]. By providing organisational support and prioritizing the care manager function both knowledge and motivation will grow, allowing the function to become an integrated part of the PCC. Ultimately, this will promote personnel well-being and improve patient care quality [4,19,37].

## Conclusions

The findings suggest that having a care manager on site in the PCC may have had limited impact on the individual work of PCC personnel, or their perceptions of the work environment, However, increased knowledge of the function appears to strengthen motivation to collaborate with the care manager and contribute to improved support among colleagues within the PCC. These observed changes suggest possible positive changes in the social and organisational work environment, although more data points are needed to confirm this development. Variations over time and between professional groups highlight the need for ongoing knowledge dissemination and tailored support to ensure sustainable and equitable benefits across the primary care work environment.

## Supplementary Material

Supplementary File 2 Organisational Work Enviroment.docx

Supplementary File 3 Social Work Enviroment.docx

Supplementary File 1 Questionary.pdf

## Data Availability

The datasets used and analysed during the current study are available from the corresponding author on reasonable request. The data are not public available due to privacy and ethical restrictions.

## List of abbreviations

CMD: Common mental disorders
PCC: Primary Care Center
PCMQ I: Implementation of care managers for patients with depression: a cross-sectional study in Swedish primary care
