# Supplementary material for: Organisational and social work-environment experiences after care manager implementation: a repeated cross-sectional study in Swedish primary care
Source: Scand J Prim Health Care. 2025 Jul 28;44(1):1–12. doi: 10.1080/02813432.2025.2538486 (PMC12918284; doi:10.1080/02813432.2025.2538486)
Supplement: Supplementary File 2 Organisational Work Enviroment.docx [file IPRI_A_2538486_SM2922.docx]

|  |  |  |  |  |  |  |  |
| --- | --- | --- | --- | --- | --- | --- | --- |
| CFIR-Conctruct | | Year | Completely/ partly disagree | Neither agree nor disagree | Almost/ completely agree | Change | P Value* |
| **Domain 2: Outer Setting** | | | % | % | % |  |  |
| *Patient needs and resources* | | | | | | | |
| Working with care manager has low priority | | | | | | | |
| Profession | Clinicans | 2016/17 | 67.8 | 15.9 | 16.3 |  |  |
|  |  | 2021/22 | 57.7 | 26.2 | 16.1 | Negative | 0.038 |
|  | Administrative | 2016/17 | 66.7 | 26.7 | 6.7 |  |  |
|  |  | 2021/22 | 47.1 | 41.2 | 11.8 | Positive | NS |
| Gender | *Woman* | 2016/17 | 67.2 | 18.1 | 14.7 |  |  |
|  |  | 2021/22 | 57.7 | 28.2 | 14.1 | Positive | NS |
|  | *Men* | 2016/17 | 67.3 | 14.3 | 18.4 |  |  |
|  |  | 2021/22 | 53.6 | 25,0 | 21.4 | Positive | NS |
| Employment | *Private* | 2016/17 | 74.4 | 12.2 | 13.3 |  |  |
|  |  | 2021/22 | 65.8 | 28.9 | 5.3 | Positive | NS |
|  | *Public* | 2016/17 | 63.8 | 19.6 | 16.6 |  |  |
|  |  | 2021/22 | 54.7 | 27.3 | 18.0 | Positive | NS |
| Age | *20-30* | 2016/17 | 69.6 | 17.4 | 13.0 |  |  |
|  |  | 2021/22 | 53.3 | 46.7 | 0.0 | Positive | NS |
|  | *31-50* | 2016/17 | 63.8 | 21.1 | 15.1 |  |  |
|  |  | 2021/22 | 50.0 | 28.4 | 21.6 | Positive | NS |
|  | 51- | 2016/17 | 70.8 | 12.4 | 16.8 |  |  |
|  |  | 2021/22 | 66.2 | 23,0 | 10.8 | Positive | NS |
| PCC size | *Small* | 2016/17 | 66.2 | 20.3 | 13.5 |  |  |
|  |  | 2021/22 | 53.6 | 28.6 | 17.9 | Negative | NS |
|  | *Large* | 2016/17 | 70.3 | 14.6 | 15.1 |  |  |
|  |  | 2021/22 | 59.3 | 28.1 | 12.6 | Positive | 0.014 |
| Geography | *Countryside* | 2016/17 | 63.1 | 23.1 | 13.8 |  |  |
|  |  | 2021/22 | 60.0 | 24.0 | 16.0 | Positive | NS |
|  | *Urban* | 2016/17 | 70.6 | 15.6 | 13.8 |  |  |
|  |  | 2021/22 | 57.7 | 29.6 | 12.7 | Positive | NS |
|  | *City* | 2016/17 | 71.8 | 11.8 | 16.5 |  |  |
|  |  | 2021/22 | 58.2 | 28.4 | 13.4 | Positive | 0.036 |
|  |  |  |  |  |  |  |  |
|  | | Year | Completely/ partly disagree | Neither agree nor disagree | Almost/ completely agree | Change | P Value* |
|  | | | % | % | % |  |  |
| The care manager's function has led to a noticeable change in my work with treating patients | | | | | | | |
| Profession | Clinicans | 2016/17 | 30.6 | 26.7 | 42.7 |  |  |
|  |  | 2021/22 | 32.2 | 30.2 | 37.6 | Negative | NS |
|  | Administrative | 2016/17 | 50.0 | 21.4 | 28.6 |  |  |
|  |  | 2021/22 | 41.2 | 35.3 | 23.5 | Negative | NS |
| Gender | *Woman* | 2016/17 | 34.7 | 27.4 | 37.9 |  |  |
|  |  | 2021/22 | 33.8 | 27.0 | 39.2 | Positive | NS |
|  | *Men* | 2016/17 | 23.5 | 23.5 | 52.9 |  |  |
|  |  | 2021/22 | 28.6 | 46.4 | 25.0 | Negative | 0.04 |
| Employment | *Private* | 2016/17 | 28.4 | 25.3 | 46.3 |  |  |
|  |  | 2021/22 | 36.8 | 23.7 | 39.5 | Negative | NS |
|  | *Public* | 2016/17 | 34,0 | 26.9 | 39.2 |  |  |
|  |  | 2021/22 | 31.9 | 31.9 | 36.2 | Negative | NS |
| Age | *20-30* | 2016/17 | 26.1 | 21.7 | 52.2 |  |  |
|  |  | 2021/22 | 33.3 | 40.0 | 26.7 | Negative | NS |
|  | *31-50* | 2016/17 | 35.6 | 23.8 | 40.6 |  |  |
|  |  | 2021/22 | 39.8 | 27.3 | 33.0 | Negative | NS |
|  | 51- | 2016/17 | 29.0 | 30.6 | 40.3 |  |  |
|  |  | 2021/22 | 24.7 | 31.5 | 43.8 | Positive | NS |
| PCC size | *Small* | 2016/17 | 35.4 | 20.3 | 44.3 |  |  |
|  |  | 2021/22 | 17.9 | 42.9 | 39.3 | Negative | NS |
|  | *Large* | 2016/17 | 29.1 | 29.1 | 41.8 |  |  |
|  |  | 2021/22 | 34.3 | 28.4 | 37.3 | Negative | NS |
| Geography | *Countryside* | 2016/17 | 28.6 | 30.2 | 41.3 |  |  |
|  |  | 2021/22 | 32.0 | 28.0 | 40.0 | Negative | NS |
|  | *Urban* | 2016/17 | 31.7 | 23.3 | 45.0 |  |  |
|  |  | 2021/22 | 35.7 | 22.9 | 41.4 | Negative | NS |
|  | *City* | 2016/17 | 31.5 | 28.3 | 40.2 |  |  |
|  |  | 2021/22 | 26.9 | 40.3 | 32.8 | Negative | NS |
|  |  |  |  |  |  |  |  |
|  | | Year | Completely/ partly disagree | Neither agree nor disagree | Almost/ completely agree | Change | P Value* |
|  | | | % | % | % |  |  |
| *External policies and incentives* | | | | | | | |
| Working with care manager has high priority | | | | | | | |
| Profession | Clinicans | 2016/17 | 11.6 | 26.5 | 61.8 |  |  |
|  |  | 2021/22 | 32.2 | 22.1 | 45.6 | Negative | <.001 |
|  | Administrative | 2016/17 | 33.3 | 16.7 | 50.0 |  |  |
|  |  | 2021/22 | 23.5 | 47.1 | 29.4 | Positive | NS |
| Gender | *Woman* | 2016/17 | 13.9 | 27.4 | 58.7 |  |  |
|  |  | 2021/22 | 32.2 | 22.8 | 45.0 | Negative | <.001 |
|  | *Men* | 2016/17 | 11.1 | 24.1 | 64.8 |  |  |
|  |  | 2021/22 | 21.4 | 35.7 | 42.9 | Negative | NS |
| Employment | *Private* | 2016/17 | 7.0 | 22.1 | 70.9 |  |  |
|  |  | 2021/22 | 31.6 | 23.7 | 44.7 | Negative | <.001 |
|  | *Public* | 2016/17 | 15.1 | 28.3 | 56.6 |  |  |
|  |  | 2021/22 | 30.2 | 25.2 | 44.6 | Negative | 0.003 |
| Age | *20-30* | 2016/17 | 9.1 | 18.2 | 72.7 |  |  |
|  |  | 2021/22 | 26.7 | 26.7 | 46.7 | Negative | NS |
|  | *31-50* | 2016/17 | 14.6 | 28.5 | 57.0 |  |  |
|  |  | 2021/22 | 38.6 | 23.9 | 37.5 | Negative | <.001 |
|  | 51- | 2016/17 | 11.7 | 24.3 | 64.0 |  |  |
|  |  | 2021/22 | 21.6 | 25.7 | 52.7 | Negative | NS |
| PCC size | *Small* | 2016/17 | 14.1 | 21.8 | 64.1 |  |  |
|  |  | 2021/22 | 28.6 | 28.6 | 42.9 | Negative | NS |
|  | *Large* | 2016/17 | 12.6 | 25.7 | 61.7 |  |  |
|  |  | 2021/22 | 28.9 | 24.4 | 46.7 | Negative | 0.001 |
| Geography | *Countryside* | 2016/17 | 14.1 | 25.0 | 60.9 |  |  |
|  |  | 2021/22 | 32.0 | 24.0 | 44.0 | Negative | NS |
|  | *Urban* | 2016/17 | 14.3 | 27.7 | 58.0 |  |  |
|  |  | 2021/22 | 29.6 | 25.4 | 45.1 | Negative | 0.042 |
|  | *City* | 2016/17 | 10.6 | 20,0 | 69.4 |  |  |
|  |  | 2021/22 | 26.9 | 25.4 | 47.8 | Negative | 0.012 |
|  |  |  |  |  |  |  |  |
|  | | Year | Completely/ partly disagree | Neither agree nor disagree | Almost/ completely agree | Change | P Value* |
| **Domain 3: Inner setting** | | | % | % | % |  |  |
| *Readiness for Implementation* | | | | | | | |
| At my PHCC there are routines and/or guidelines for the care manager function | | | | | | | |
| Profession | Clinicans | 2016/17 | 13.9 | 25.1 | 61.0 |  |  |
|  |  | 2021/22 | 23.5 | 28.2 | 48.3 | Negative | 0.01 |
|  | Administrative | 2016/17 | 15.6 | 15.6 | 68.9 |  |  |
|  |  | 2021/22 | 11.8 | 41.2 | 47.1 | Negative | NS |
| Gender | *Woman* | 2016/17 | 14.8 | 24.2 | 61.0 |  |  |
|  |  | 2021/22 | 19.3 | 32,0 | 48.7 | Negative | 0.035 |
|  | *Men* | 2016/17 | 15.6 | 23.4 | 60.9 |  |  |
|  |  | 2021/22 | 28.6 | 25.0 | 46.4 | Negative | NS |
| Employment | *Private* | 2016/17 | 10.2 | 29.1 | 60.6 |  |  |
|  |  | 2021/22 | 10.5 | 28.9 | 60.5 | Positive | NS |
|  | *Public* | 2016/17 | 16.1 | 21.8 | 62.1 |  |  |
|  |  | 2021/22 | 23.6 | 31.4 | 45.0 | Negative | 0.003 |
| Age | *20-30* | 2016/17 | 12.9 | 19.4 | 67.7 |  |  |
|  |  | 2021/22 | 33.3 | 46.7 | 20.0 | Negative | 0.01 |
|  | *31-50* | 2016/17 | 17.7 | 24.3 | 58.0 |  |  |
|  |  | 2021/22 | 23.9 | 36.4 | 39.8 | Negative | 0.009 |
|  | 51- | 2016/17 | 10.8 | 24.2 | 65.1 |  |  |
|  |  | 2021/22 | 14.7 | 21.3 | 64.0 | Negative | NS |
| PCC size | *Small* | 2016/17 | 14.9 | 24.8 | 60.4 |  |  |
|  |  | 2021/22 | 13.8 | 37.9 | 48.3 | Negative | NS |
|  | *Large* | 2016/17 | 14.5 | 23.9 | 61.6 |  |  |
|  |  | 2021/22 | 21.5 | 30.4 | 48.1 | Negative | 0.027 |
| Geography | *Countryside* | 2016/17 | 11,0 | 29.3 | 59.8 |  |  |
|  |  | 2021/22 | 28,0 | 32.0 | 40.0 | Negative | NS |
|  | *Urban* | 2016/17 | 17.7 | 22.4 | 59.9 |  |  |
|  |  | 2021/22 | 20.8 | 36.1 | 43.1 | Negative | 0.035 |
|  | *City* | 2016/17 | 12.1 | 23.4 | 64.5 |  |  |
|  |  | 2021/22 | 16.4 | 26.9 | 56.7 | Negative | NS |
|  |  |  |  |  |  |  |  |
|  | | Year | Completely/ partly disagree | Neither agree nor disagree | Almost/ completely agree | Change | P Value* |
|  | | | % | % | % |  |  |
| The PHCC has a clearly formulated goal regarding the care manager function | | | | | | | |
| Profession | Clinicans | 2016/17 | 26.9 | 49.5 | 23.6 |  |  |
|  |  | 2021/22 | 23.5 | 28.2 | 48.3 | Positive | <.001 |
|  | Administrative | 2016/17 | 24.4 | 33.3 | 42.2 |  |  |
|  |  | 2021/22 | 11.8 | 41.2 | 47.1 | Positive | <.001 |
| Gender | *Woman* | 2016/17 | 27.8 | 45.3 | 27.0 |  |  |
|  |  | 2021/22 | 19.5 | 32.2 | 48.3 | Positive | <.001 |
|  | *Men* | 2016/17 | 25.0 | 56.3 | 18.8 |  |  |
|  |  | 2021/22 | 28.6 | 25.0 | 46.4 | Positive | 0.008 |
| Employment | *Private* | 2016/17 | 19,0 | 60.3 | 20.6 |  |  |
|  |  | 2021/22 | 10.5 | 28.9 | 60.5 | Positive | <.001 |
|  | *Public* | 2016/17 | 30.0 | 42.9 | 27.1 |  |  |
|  |  | 2021/22 | 23.7 | 31.7 | 44.6 | Positive | 0.001 |
| Age | *20-30* | 2016/17 | 19.4 | 58.1 | 22.6 |  |  |
|  |  | 2021/22 | 33.3 | 46.7 | 20.0 | Negative | NS |
|  | *31-50* | 2016/17 | 30.1 | 47.3 | 22.6 |  |  |
|  |  | 2021/22 | 23.9 | 36.4 | 39.8 | Positive | 0.012 |
|  | 51- | 2016/17 | 24.3 | 47,0 | 28.6 |  |  |
|  |  | 2021/22 | 14.9 | 21.6 | 63.5 | Positive | <.001 |
| PCC size | *Small* | 2016/17 | 32.0 | 52.0 | 16.0 |  |  |
|  |  | 2021/22 | 14.3 | 39.3 | 46.4 | Positive | 0.003 |
|  | *Large* | 2016/17 | 24.2 | 47.5 | 28.3 |  |  |
|  |  | 2021/22 | 21.5 | 30.4 | 48.1 | Positive | <.001 |
| Geography | *Countryside* | 2016/17 | 25.9 | 63.0 | 11.1 |  |  |
|  |  | 2021/22 | 28.0 | 32.0 | 40.0 | Positive | 0.003 |
|  | *Urban* | 2016/17 | 28.1 | 40.1 | 31.8 |  |  |
|  |  | 2021/22 | 21.1 | 36.6 | 42.3 | Positive | NS |
|  | *City* | 2016/17 | 23.4 | 52.4 | 24.2 |  |  |
|  |  | 2021/22 | 16.4 | 26.9 | 56.7 | Positive | 0.003 |
|  |  |  |  |  |  |  |  |
|  | | Year | Completely/ partly disagree | Neither agree nor disagree | Almost/ completely agree | Change | P Value* |
|  | | | % | % | % |  |  |
| Lack of clarity regarding what the care manager function entails | | | | | | | |
| Profession | Clinicans | 2016/17 | 60.6 | 15.6 | 23.8 |  |  |
|  |  | 2021/22 | 44.3 | 28.9 | 26.8 | Negative | <.001 |
|  | Administrative | 2016/17 | 71.4 | 7.1 | 21.4 |  |  |
|  |  | 2021/22 | 64.7 | 23.5 | 11.8 | Positive | NS |
| Gender | *Woman* | 2016/17 | 61.3 | 14.4 | 24.3 |  |  |
|  |  | 2021/22 | 48.3 | 26.2 | 25.5 | Positive | 0.006 |
|  | *Men* | 2016/17 | 62.3 | 17.0 | 20.8 |  |  |
|  |  | 2021/22 | 39.3 | 35.7 | 25.0 | Negative | NS |
| Employment | *Private* | 2016/17 | 68.7 | 16.2 | 15.2 |  |  |
|  |  | 2021/22 | 47.4 | 28.9 | 23.7 | Positive | NS |
|  | *Public* | 2016/17 | 58.4 | 14.3 | 27.3 |  |  |
|  |  | 2021/22 | 46.8 | 27.3 | 25.9 | Negative | 0.006 |
| Age | *20-30* | 2016/17 | 63.6 | 18.2 | 18.2 |  |  |
|  |  | 2021/22 | 40.0 | 40.0 | 20.0 | Negative | NS |
|  | *31-50* | 2016/17 | 59.4 | 16.7 | 23.9 |  |  |
|  |  | 2021/22 | 42.0 | 25.0 | 33.0 | Negative | 0.026 |
|  | 51- | 2016/17 | 63.8 | 12.1 | 24.1 |  |  |
|  |  | 2021/22 | 54.1 | 28.4 | 17.6 | Positive | <.001 |
| PCC size | *Small* | 2016/17 | 62.9 | 20.2 | 16.9 |  |  |
|  |  | 2021/22 | 53.6 | 28.6 | 17.9 | Negative | NS |
|  | *Large* | 2016/17 | 62.4 | 11.3 | 26.2 |  |  |
|  |  | 2021/22 | 44.4 | 29.6 | 25.9 | Positive | <.001 |
| Geography | *Countryside* | 2016/17 | 60,0 | 14.7 | 25.3 |  |  |
|  |  | 2021/22 | 36.0 | 28.0 | 36.0 | Negative | NS |
|  | *Urban* | 2016/17 | 58.2 | 12.7 | 29.1 |  |  |
|  |  | 2021/22 | 50.7 | 26.8 | 22.5 | Positive | 0.048 |
|  | *City* | 2016/17 | 70.3 | 14.9 | 14.9 |  |  |
|  |  | 2021/22 | 44.8 | 32.8 | 22.4 | Negative | 0.003 |
|  |  |  |  |  |  |  |  |
|  | | Year | Completely/ partly disagree | Neither agree nor disagree | Almost/ completely agree | Change | P Value* |
|  | | | % | % | % |  |  |
| Lack of clarity regarding the distribution of responsibility between myself as personal member and the care manager | | | | | | | |
| Profession | Clinicans | 2016/17 | 63.7 | 16.5 | 19.9 |  |  |
|  |  | 2021/22 | 51.0 | 22.1 | 26.8 | Positive | 0.042 |
|  | Administrative | 2016/17 | 58.3 | 33.3 | 8.3 |  |  |
|  |  | 2021/22 | 52.9 | 35.3 | 11.8 | Negative | NS |
| Gender | *Woman* | 2016/17 | 62.2 | 17.3 | 20.4 |  |  |
|  |  | 2021/22 | 53.7 | 22.1 | 24.2 | Positive | NS |
|  | *Men* | 2016/17 | 69.4 | 18.4 | 12.2 |  |  |
|  |  | 2021/22 | 53.6 | 25.0 | 21.4 | Positive | NS |
| Employment | *Private* | 2016/17 | 67.0 | 17.0 | 15.9 |  |  |
|  |  | 2021/22 | 65.8 | 18.4 | 15.8 | Positive | NS |
|  | *Public* | 2016/17 | 61.9 | 17.5 | 20.6 |  |  |
|  |  | 2021/22 | 50.4 | 23.7 | 25.9 | Positive | NS |
| Age | *20-30* | 2016/17 | 84.2 | 5.3 | 10.5 |  |  |
|  |  | 2021/22 | 33.3 | 40.0 | 26.7 | Positive | 0.008 |
|  | *31-50* | 2016/17 | 59.7 | 21.5 | 18.8 |  |  |
|  |  | 2021/22 | 50,0 | 22.7 | 27.3 | Positive | NS |
|  | 51- | 2016/17 | 64.9 | 14.0 | 21.1 |  |  |
|  |  | 2021/22 | 62.2 | 18.9 | 18.9 | Positive | NS |
| PCC size | *Small* | 2016/17 | 69,0 | 15.5 | 15.5 |  |  |
|  |  | 2021/22 | 39.3 | 42.9 | 17.9 | Positive | 0.011 |
|  | *Large* | 2016/17 | 62.7 | 17.3 | 20.0 |  |  |
|  |  | 2021/22 | 56.3 | 19.3 | 24.4 | Positive | NS |
| Geography | *Countryside* | 2016/17 | 65.1 | 14.3 | 20.6 |  |  |
|  |  | 2021/22 | 52.0 | 16.0 | 32.0 | Positive | NS |
|  | *Urban* | 2016/17 | 61.3 | 18.0 | 20.7 |  |  |
|  |  | 2021/22 | 53.5 | 23.9 | 22.5 | Positive | NS |
|  | *City* | 2016/17 | 68.3 | 17.1 | 14.6 |  |  |
|  |  | 2021/22 | 53.7 | 25.4 | 20.9 | Positive | NS |
|  |  |  |  |  |  |  |  |
| CFIR. Consolidated Framework for Implementation Research; NS. Non significant. *Significant difference p=<0.005 | | | | | | | |
