# Supplementary material for: Organisational and social work-environment experiences after care manager implementation: a repeated cross-sectional study in Swedish primary care
Source: Scand J Prim Health Care. 2025 Jul 28;44(1):1–12. doi: 10.1080/02813432.2025.2538486 (PMC12918284; doi:10.1080/02813432.2025.2538486)
Supplement: Supplementary File 3 Social Work Enviroment.docx [file IPRI_A_2538486_SM2921.docx]

|  |  |  |  |  |  |  |  |
| --- | --- | --- | --- | --- | --- | --- | --- |
| CFIR-Conctruct | | Year | Completely/ partly disagree | Neither agree nor disagree | Almost/ completely agree | Change | P Value* |
| **Domain 3: Inner setting** | | | % | % | % |  |  |
| *Networks and communications* | | | | | | | |
| I can collaborate with the care manager without encountering any problems | | | | | | | |
| Profession | Clinicans | 2016/17 | 11.3 | 12.0 | 76.8 |  |  |
|  |  | 2021/22 | 14.8 | 19.5 | 65.8 | Negative | 0.043 |
|  | Administrative | 2016/17 | 17.6 | 17.6 | 64.7 |  |  |
|  |  | 2021/22 | 5.9 | 52.9 | 41.2 | Negative | NS |
| Gender | *Woman* | 2016/17 | 13.3 | 13.3 | 73.4 |  |  |
|  |  | 2021/22 | 14.1 | 23.5 | 62.4 | Negative | 0.026 |
|  | *Men* | 2016/17 | 6.0 | 8.0 | 86.0 |  |  |
|  |  | 2021/22 | 10.7 | 21.4 | 67.9 | Negative | NS |
| Employment | *Private* | 2016/17 | 7.6 | 7.6 | 84.8 |  |  |
|  |  | 2021/22 | 5.3 | 23.7 | 71.1 | Negative | 0.046 |
|  | *Public* | 2016/17 | 14.1 | 14.1 | 71.8 |  |  |
|  |  | 2021/22 | 15.8 | 23.0 | 61.2 | Negative | NS |
| Age | *20-30* | 2016/17 | 0.0 | 4.8 | 95.2 |  |  |
|  |  | 2021/22 | 20.0 | 13.3 | 66.7 | Negative | NS |
|  | *31-50* | 2016/17 | 11.0 | 11.7 | 77.3 |  |  |
|  |  | 2021/22 | 14.8 | 27.3 | 58.0 | Negative | 0.003 |
|  | 51- | 2016/17 | 15.7 | 14.0 | 70.2 |  |  |
|  |  | 2021/22 | 10.8 | 20.3 | 68.9 | Negative | 0.004 |
| PCC size | *Small* | 2016/17 | 14.1 | 14.1 | 71.8 |  |  |
|  |  | 2021/22 | 7.1 | 42.9 | 50.0 | Negative | 0.008 |
|  | *Large* | 2016/17 | 12.3 | 10.8 | 76.9 |  |  |
|  |  | 2021/22 | 13.3 | 20.0 | 66.7 | Negative | NS |
| Geography | *Countryside* | 2016/17 | 11.3 | 9.7 | 79.0 |  |  |
|  |  | 2021/22 | 32.0 | 12.0 | 56.0 | Negative | 0.047 |
|  | *Urban* | 2016/17 | 17.4 | 14,0 | 68.6 |  |  |
|  |  | 2021/22 | 14.1 | 23.9 | 62.0 | Negative | NS |
|  | *City* | 2016/17 | 7.8 | 10.0 | 82.2 |  |  |
|  |  | 2021/22 | 3.0 | 28.4 | 68.7 | Negative | 0.007 |
|  |  |  |  |  |  |  |  |
|  | | Year | Completely/ partly disagree | Neither agree nor disagree | Almost/ completely agree | Change | P Value* |
|  | | | % | % | % |  |  |
| I have support from colleagues regarding my cooperation with the care manager | | | | | | | |
| Profession | Clinicans | 2016/17 | 9.7 | 19.4 | 70.9 |  |  |
|  |  | 2021/22 | 16.8 | 18.1 | 65.1 | Negative | NS |
|  | Administrative | 2016/17 | 11.1 | 11.1 | 77.8 |  |  |
|  |  | 2021/22 | 17.6 | 41.2 | 41.2 | Negative | NS |
| Gender | *Woman* | 2016/17 | 9.6 | 19.7 | 70.7 |  |  |
|  |  | 2021/22 | 18.1 | 20.1 | 61.7 | Negative | NS |
|  | *Men* | 2016/17 | 13.6 | 20.5 | 65.9 |  |  |
|  |  | 2021/22 | 7.1 | 21.4 | 71.4 | Positive | NS |
| Employment | *Private* | 2016/17 | 4.8 | 15.7 | 79.5 |  |  |
|  |  | 2021/22 | 10.5 | 21.1 | 68.4 | Negative | NS |
|  | *Public* | 2016/17 | 13.1 | 21.0 | 65.9 |  |  |
|  |  | 2021/22 | 18,0 | 20.1 | 61.9 | Negative | NS |
| Age | *20-30* | 2016/17 | 10.6 | 14.9 | 74.5 |  |  |
|  |  | 2021/22 | 18.2 | 26.1 | 55.7 | Negative | NS |
|  | *31-50* | 2016/17 | 11.5 | 25.0 | 63.5 |  |  |
|  |  | 2021/22 | 13.5 | 12.2 | 74.3 | Positive | 0.012 |
|  | 51- | 2016/17 | 10.4 | 19.3 | 70.3 |  |  |
|  |  | 2021/22 | 16.4 | 20.3 | 63.3 | Negative | NS |
| PCC size | *Small* | 2016/17 | 15.9 | 14.3 | 69.8 |  |  |
|  |  | 2021/22 | 3.6 | 42.9 | 53.6 | Negative | 0.009 |
|  | *Large* | 2016/17 | 8.8 | 21.2 | 70.0 |  |  |
|  |  | 2021/22 | 18.5 | 15.6 | 65.9 | Negative | 0.033 |
| Geography | *Countryside* | 2016/17 | 10.9 | 18.2 | 70.9 |  |  |
|  |  | 2021/22 | 40.0 | 8.0 | 52.0 | Negative | 0.012 |
|  | *Urban* | 2016/17 | 13.3 | 22.9 | 63.8 |  |  |
|  |  | 2021/22 | 16.9 | 21.1 | 62.0 | Negative | NS |
|  | *City* | 2016/17 | 6.8 | 15.1 | 78.1 |  |  |
|  |  | 2021/22 | 6.0 | 23.9 | 70.1 | Negative | NS |
|  |  |  |  |  |  |  |  |
|  | | Year | Completely/ partly disagree | Neither agree nor disagree | Almost/ completely agree | Change | P Value* |
|  | | | % | % | % |  |  |
| I have support from the PHCC director regarding my cooperation with the care manager | | | | | | | |
| Profession | Clinicans | 2016/17 | 5.3 | 15.8 | 78.9 |  |  |
|  |  | 2021/22 | 18.8 | 17.4 | 63.8 | Negative | <.001 |
|  | Administrative | 2016/17 | 0.0 | 18.2 | 81.8 |  |  |
|  |  | 2021/22 | 17.6 | 47.1 | 35.3 | Negative | NS |
| Gender | *Woman* | 2016/17 | 4.5 | 17.0 | 78.6 |  |  |
|  |  | 2021/22 | 18.8 | 21.5 | 59.7 | Negative | <.001 |
|  | *Men* | 2016/17 | 10.2 | 12.2 | 77.6 |  |  |
|  |  | 2021/22 | 14.3 | 14.3 | 71.4 | Negative | NS |
| Employment | *Private* | 2016/17 | 6.9 | 6.9 | 86.2 |  |  |
|  |  | 2021/22 | 10.5 | 21.1 | 68.4 | Negative | 0.041 |
|  | *Public* | 2016/17 | 5.2 | 19.6 | 75.3 |  |  |
|  |  | 2021/22 | 20.1 | 20.1 | 59.7 | Negative | <.001 |
| Age | *20-30* | 2016/17 | 4.8 | 9.5 | 85.7 |  |  |
|  |  | 2021/22 | 20.0 | 20.0 | 60.0 | Negative | NS |
|  | *31-50* | 2016/17 | 4.6 | 15.2 | 80.1 |  |  |
|  |  | 2021/22 | 22.7 | 21.6 | 55.7 | Negative | <.001 |
|  | 51- | 2016/17 | 7.3 | 17.4 | 75.2 |  |  |
|  |  | 2021/22 | 12.2 | 18.9 | 68.9 | Negative | NS |
| PCC size | *Small* | 2016/17 | 7.4 | 16.2 | 76.5 |  |  |
|  |  | 2021/22 | 3.6 | 42.9 | 53.6 | Negative | 0.025 |
|  | *Large* | 2016/17 | 4.4 | 14.4 | 81.1 |  |  |
|  |  | 2021/22 | 20.0 | 15.6 | 64.4 | Negative | <.001 |
| Geography | *Countryside* | 2016/17 | 5.4 | 14.3 | 80.4 |  |  |
|  |  | 2021/22 | 40.0 | 8.0 | 52.0 | Negative | <.001 |
|  | *Urban* | 2016/17 | 6.4 | 20.0 | 73.6 |  |  |
|  |  | 2021/22 | 18.3 | 22.5 | 59.2 | Negative | 0.033 |
|  | *City* | 2016/17 | 3.7 | 8.5 | 87.8 |  |  |
|  |  | 2021/22 | 7.5 | 22.4 | 70.1 | Negative | 0.03 |
|  |  |  |  |  |  |  |  |
|  | | Year | Completely/ partly disagree | Neither agree nor disagree | Almost/ completely agree | Change | P Value* |
| **Domain 4: Characteristics of Individuals** | | | % | % | % |  |  |
| *Knowledge and beliefs of the Intervention* | | | | | | | |
| I have sufficent knowledge about the care manager function | | | | | | | |
| Profession | Clinicans* | 2016/17 | 30.5 | 19.4 | 50.1 |  |  |
|  |  | 2021/22 | 40.3 | 24.8 | 34.9 | Negative | 0.006 |
|  | Administrative | 2016/17 | 39.1 | 26.1 | 34.8 |  |  |
|  |  | 2021/22 | 47.1 | 35.3 | 17.6 | Negative | NS |
| Gender | *Woman* | 2016/17 | 35.3 | 20.9 | 43.9 |  |  |
|  |  | 2021/22 | 43.6 | 25.5 | 30.9 | Negative | 0.024 |
|  | *Men* | 2016/17 | 15.6 | 15.6 | 68.8 |  |  |
|  |  | 2021/22 | 28.6 | 25.0 | 46.4 | Negative | NS |
| Employment | *Private* | 2016/17 | 26.4 | 14.0 | 59.7 |  |  |
|  |  | 2021/22 | 36.8 | 26.3 | 36.8 | Negative | 0.037 |
|  | *Public* | 2016/17 | 34.3 | 22.3 | 43.4 |  |  |
|  |  | 2021/22 | 42.4 | 25.2 | 32.4 | Negative | NS |
| Age | *20-30* | 2016/17 | 34.4 | 15.6 | 50.0 |  |  |
|  |  | 2021/22 | 60.0 | 26.7 | 13.3 | Negative | 0.047 |
|  | *31-50* | 2016/17 | 31.0 | 20.4 | 48.7 |  |  |
|  |  | 2021/22 | 40.9 | 25.0 | 34.1 | Negative | NS |
|  | 51- | 2016/17 | 33,0 | 20.2 | 46.8 |  |  |
|  |  | 2021/22 | 37.8 | 25.7 | 36.5 | Negative | NS |
| PCC size | *Small* | 2016/17 | 32.4 | 17.6 | 50.0 |  |  |
|  |  | 2021/22 | 25.0 | 46.4 | 28.6 | Negative | 0.01 |
|  | *Large* | 2016/17 | 32,0 | 20.7 | 47.3 |  |  |
|  |  | 2021/22 | 43.7 | 20.7 | 35.6 | Negative | 0.04 |
| Geography | *Countryside* | 2016/17 | 26.8 | 17.1 | 56.1 |  |  |
|  |  | 2021/22 | 44.0 | 12.0 | 44.0 | Negative | NS |
|  | *Urban* | 2016/17 | 36.1 | 18.0 | 45.9 |  |  |
|  |  | 2021/22 | 50.7 | 22.5 | 26.8 | Negative | 0.016 |
|  | *City* | 2016/17 | 29.4 | 24.6 | 46.0 |  |  |
|  |  | 2021/22 | 28.4 | 32.8 | 38.8 | Negative | NS |
|  |  |  |  |  |  |  |  |
|  | | Year | Completely/ partly disagree | Neither agree nor disagree | Almost/ completely agree | Change | P Value* |
|  | | | % | % | % |  |  |
| I am motivated to cooperate with the care manager at my PHCC | | | | | | | |
| Profession | Clinicans | 2016/17 | 7.5 | 76.4 | 15.1 |  |  |
|  |  | 2021/22 | 8.1 | 10.7 | 21.2 | Positive | <.001 |
|  | Administrative | 2016/17 | 8.9 | 55.6 | 19.1 |  |  |
|  |  | 2021/22 | 0.0 | 23.5 | 20.2 | Positive | 0.018 |
| Gender | *Woman* | 2016/17 | 8.8 | 72.7 | 18.1 |  |  |
|  |  | 2021/22 | 7.4 | 13.4 | 24.2 | Positive | <.001 |
|  | *Men* | 2016/17 | 3.1 | 81.3 | 11.1 |  |  |
|  |  | 2021/22 | 7.1 | 7.1 | 21.2 | Positive | <.001 |
| Employment | *Private* | 2016/17 | 7.0 | 77.5 | 10.1 |  |  |
|  |  | 2021/22 | 5.3 | 7.9 | 23.2 | Positive | <.001 |
|  | *Public* | 2016/17 | 8.2 | 73.3 | 12.1 |  |  |
|  |  | 2021/22 | 7.9 | 13.7 | 29.2 | Positive | <.001 |
| Age | *20-30* | 2016/17 | 0.0 | 81.3 | 10.1 |  |  |
|  |  | 2021/22 | 13.3 | 20.0 | 23.2 | Positive | <.001 |
|  | *31-50* | 2016/17 | 6.6 | 77.9 | 16.1 |  |  |
|  |  | 2021/22 | 6.8 | 13.6 | 28.2 | Positive | <.001 |
|  | 51- | 2016/17 | 10.6 | 69.1 | 16.1 |  |  |
|  |  | 2021/22 | 6.8 | 9.5 | 28.2 | Positive | <.001 |
| PCC size | *Small* | 2016/17 | 7.9 | 81.2 | 19.1 |  |  |
|  |  | 2021/22 | 0.0 | 14.3 | 20.2 | Positive | <.001 |
|  | *Large* | 2016/17 | 7.3 | 72.7 | 13.1 |  |  |
|  |  | 2021/22 | 7.4 | 12.6 | 24.2 | Positive | <.001 |
| Geography | *Countryside* | 2016/17 | 7.3 | 84.1 | 13.1 |  |  |
|  |  | 2021/22 | 24.0 | 16.0 | 20.2 | Positive | <.001 |
|  | *Urban* | 2016/17 | 8.8 | 68.4 | 18.1 |  |  |
|  |  | 2021/22 | 2.8 | 15.5 | 28.2 | Positive | <.001 |
|  | *City* | 2016/17 | 5.6 | 78.6 | 16.1 |  |  |
|  |  | 2021/22 | 3.0 | 9.0 | 20.2 | Positive | <.001 |
|  |  |  |  |  |  |  |  |
| CFIR. Consolidated Framework for Implementation Research; NS. Non significant. *Significant difference p=<0.005 | | | | | | | |
