# Supplementary material for: Organisational and social work-environment experiences after care manager implementation: a repeated cross-sectional study in Swedish primary care
Source: Scand J Prim Health Care. 2025 Jul 28;44(1):1–12. doi: 10.1080/02813432.2025.2538486 (PMC12918284; doi:10.1080/02813432.2025.2538486)
Supplement: Supplementary File 1 Questionary.pdf [file IPRI_A_2538486_SM2920.pdf]

Invitation to Participate in a Web-Based Survey on the Influence of Care Managers for Common Mental Health on the Work Environment at Primary Care Centers

As part of the methodological development of the care manager function for common mental health in primary care, we aim to follow up on the survey conducted at your primary care center approximately five years ago with a new survey. This survey is directed at primary care center personnel and aims to gather knowledge about how the care manager function for mental health has functioned at your primary care center, with a particular focus on the social and organizational work environment. This knowledge is essential for further developing the care manager function within Swedish primary care.

Since your primary care center has a care manager for common mental health, you are invited, as a personnel member, to participate in this survey. Participation is voluntary, and you may withdraw at any time. You can take part in the study by answering the questions below.

Thank you for your participation!

/for the scientific project Care Manager

Pia Augustsson, Ph.D student
General Practice / Family Medicine, School of Public Health and Community Medicine, University of Gothenburg, Sweden

Phone: 0700-85 28 60
E-mail: pia.augustsson@gu.se

Ansvariga för studien

Cecilia Björkelund
Senior professor. General Practice / Family Medicine, School of Public Health and Community Medicine, University of Gothenburg
e-mail: cecilia.bjorkelund@allmed.gu.se

Carl Wikberg
Ph.D. General Practice / Family Medicine, School of Public Health and Community Medicine, University of Gothenburg
E-mail: carl.wikberg@allmed.gu.se

Eva-Lisa Petersson
Docent, FoUUi Primärvård Göteborg och Södra Bohuslän, Västra Götalandsregionen E-post: eva-lisa.petersson@vgregion.se

Elisabeth Björk Brämberg
Docent, Karolinska Institutet, Institutet för miljömedicin

E-post: elisabeth.bjork.bramberg@ki.se

Sven Kylén
PhD, FoUUi Primärvård Fyrbodal, Västra Götalandsregionen
E-post: sven.kylen@vgregion.se.

In this survey, we address you as a personnel at a primary care center that have a care manager for common mental health. Our questions focus on your collaboration with the care manager as a function, not as an individual, as well as your organizational and social work environment today and during the COVID-19 pandemic.

Workplace (The name of the Primary Care Center): ________________________________

Employment:
- ☐ Private
- ☐ Public

Age:
- ☐ 20–30 year
- ☐ 31–50 year
- ☐ 51 and older

Sex:
- ☐ Woman
- ☐ Male

What is your professional title?

- ☐ Occupational therapist
- ☐ District nurse
- ☐ Physiotherapist
- ☐ Physician
- ☐ Psychologist
- ☐ Coordinator of rehabilitation
- ☐ Nurse
- ☐ Psychotherapist/social worker
- ☐ Assistant nurse
- ☐ Other ________________________

Since 2015, there has been a care manager function for common mental health at primary care centers. Are you familiar with the care manager function? The care manager provides support for patients with depression and anxiety at the primary care center, maintains contact with patients via telephone, collaborates with doctors, psychologists, and other healthcare providers, and identifies issues in the care process to promote high-quality care for patients with mental health conditions.

□ Yes, I am well informed
□Yes, I am familiar with the role

□ No, but I have heard of the care manager function.
□ No, I am not familiar with the care manager function.

GÖTEBORGS UNIVERSITET

VÄSTRA GÖTALANDSREGIONEN

Questions about your knowledge and collaboration with the care manager at your primary care center.

	Completely disagree 1	2	3	4	Completely agree 5
At my PCC there are routines and/or guidelines for the care manager function	☐	☐	☐	☐	☐
The PCC has a clearly formulated goal regarding the care manager function	☐	☐	☐	☐	☐
I am motivated to collaborate with the care manager at my PCC	☐	☐	☐	☐	☐
The care manager's function has led to a noticeable change in my work with treating patients	☐	☐	☐	☐	☐
I can collaborate with the care manager without encountering any problems	☐	☐	☐	☐	☐
I have support from the PCC director regarding my cooperation with the care manager	☐	☐	☐	☐	☐
I have support from colleagues regarding my cooperation with the care manager	☐	☐	☐	☐	☐
Working with care coordination has high priority	☐	☐	☐	☐	☐
Lack of clarity regarding what the care manager function entails.	☐	☐	☐	☐	☐
Lack of clarity regarding the distribution of responsibility between myself as a personal member and the care manager.	☐	☐	☐	☐	☐
Working with care manager has low priority.	☐	☐	☐	☐	☐

GÖTEBORGS UNIVERSITET

VÄSTRA GÖTALANDSREGIONEN

Some questions about your work environment during COVID-19.

When answering these questions, please take a moment to reflect on what it was like to work during the past year, autumn/spring 2020/21.

Your Social Work Environment

How are you affected by the people around you, including social interactions, collaboration, and social support from directors and colleagues?

**To what extent do you experience that:**	To a very small extent	To some extent	Neither nor	To some extent	To a great extent
During COVID-19, there has been support for your work situation from your director	☐	☐	☐	☐	☐
During COVID-19, there has been support for your work situation among your colleagues	☐	☐	☐	☐	☐
COVID-19 has caused a deterioration in your social work environment at the primary care center	☐	☐	☐	☐	☐
COVID-19 has contributed to an improvement in your social work environment at the primary care center	☐	☐	☐	☐	☐

Rate the extent to which COVID-19 has contributed to a changed social work environment for you at the primary care center.

	1	2	3	4	5	6	7	8	9	10	
Definitely a deterioration	☐	☐	☐	☐	☐	☐	☐	☐	☐	☐	Definitely an improvement

Your organisational work environment

What resources and authorities are available to perform the work and what demands are placed on you and your colleagues

**To what extent do you experience that:**	To a very small extent	To some extent	Neither nor	To some extent	To a great extent
During COVID-19, there has been a balance between resources (time, staff) and demands (tasks, responsibilities) during your workday at the healthcare center.	☐	☐	☐	☐	☐
COVID-19 has caused a deterioration in your organisational work environment at the healthcare center.	☐	☐	☐	☐	☐
COVID-19 has contributed to an improvement in your organizational work environment at the primary care center.	☐	☐	☐	☐	☐

Rate the extent to which COVID-19 has contributed to a changed organizational work environment for you at the primary care center

	1	2	3	4	5	6	7	8	9	10	
Definitely a deterioration	☐	☐	☐	☐	☐	☐	☐	☐	☐	☐	Definitely an improvement

GÖTEBORGS UNIVERSITET

VÄSTRA GÖTALANDSREGIONEN

Care Manager

To what extent has COVID-19 led to the care manager function
☐ Has been paused in favor of somatic clinical work
☐ Has been allocated more time for work with mental health
☐ Has remained unchanged
☐ Has been allocated less time for work with mental health

To what extent do you experience that COVID-19 has made your work with the caremanager more difficult?
☐ To a very small extent
☐ To some extent
☐ Neither nor
☐ Some extent
☐ To a great extent

Rate the extent to which COVID-19 has contributed to a changed collaboration with care manager for common mental health at the primary care center.

	1	2	3	4	5	6	7	8	9	10	
Definitely a deterioration	☐	☐	☐	☐	☐	☐	☐	☐	☐	☐	Definitely an improvement

Thank you for your participation!
